# Isolated Cardiac Metastasis from Colorectal Cancer in a 35-Year-Old Man

**DOI:** 10.1155/2012/751761

**Published:** 2012-03-04

**Authors:** Jacopo Pizzicannella, Vincenzo Ricci, Riccardo Gorla, Elena Spinapolice, Antonio Esposito

**Affiliations:** ^1^Cardio-Thoracic and Vascular Department, San Raffaele Hospital, 20132 Milan, Italy; ^2^Department of Oncology, San Raffaele Hospital, 20132 Milan, Italy; ^3^Department of Nuclear Medicine, San Raffaele Hospital, 20132 Milan, Italy; ^4^Department of Radiology, San Raffaele Hospital, 20132 Milan, Italy

## Abstract

We present the case of a 35-year-old patient who was found to be affected by an isolated clinically silent cardiac metastasis despite a negative CT follow-up at one year from abdominal surgery for colorectal cancer. CT/PET and tumor marker GICA were fundamental in suggesting the diagnosis, which was then confirmed by cardiac magnetic resonance and surgical biopsy. This is a very rare modality of presentation of cardiac metastasis because of the young age of our patient and the absence of disease in other sites.

## 1. Introduction

Colorectal cancer is the third most commonly diagnosed cancer in males and the second in females with over 1,2 million new cancer cases and 608,700 deaths estimated to have occurred in 2008. Metastases from colorectal cancer can occur either by lymphatic or hematogenous spreading, and the sites most commonly involved are lymph nodes, liver, and lung. Unusual sites such as the spleen, thyroid gland, spermatic cord, and skeletal muscle have been reported as a distinctive feature of end-stage disease [1]. Cardiac metastasization from colorectal cancer is an extremely rare event and occurs either with the concomitant involvement of other organs or, more rarely, as the only localization. We report the case of a 35-year-old man who was found to be affected by an isolated clinically silent cardiac metastasis despite a negative CT follow-up at one year from abdominal surgery for colorectal cancer.

## 2. Case Presentation

A progressive increase of tumor marker GICA (180 U/mL) was detected in an asymptomatic 35-year-old patient with a history of metastatic colorectal cancer, despite a negative CT follow-up at one year from abdominal surgery. He had been treated with right hemicolectomy and liver metastasectomy followed by adjuvant chemotherapy with FOLFIRI (leucovorin, fluorouracil, and irinotecan) plus bevacizumab.

**Figure 1:**
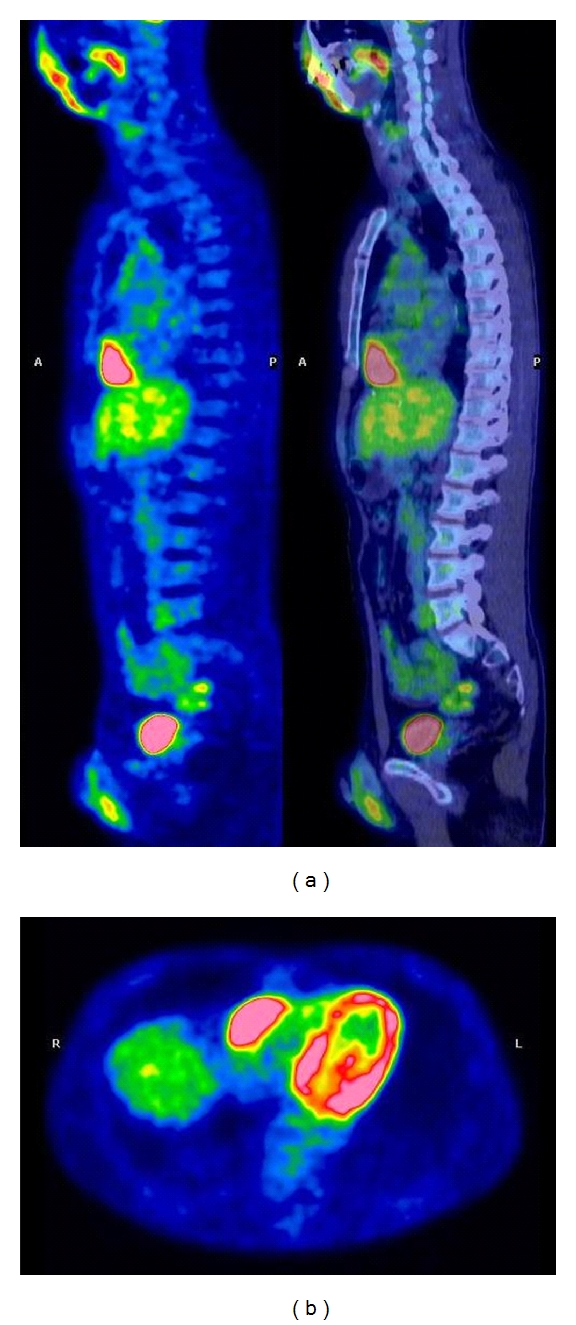
Positron emission tomography/computed tomography (PET/CT) with (18)F-fluoro-2-deoxyglucose showed an intense tracer uptake localized at the right cardiofrenic angle, as shown in transaxial and sagittal views ((b) and (a), resp.).

**Figure 2:**
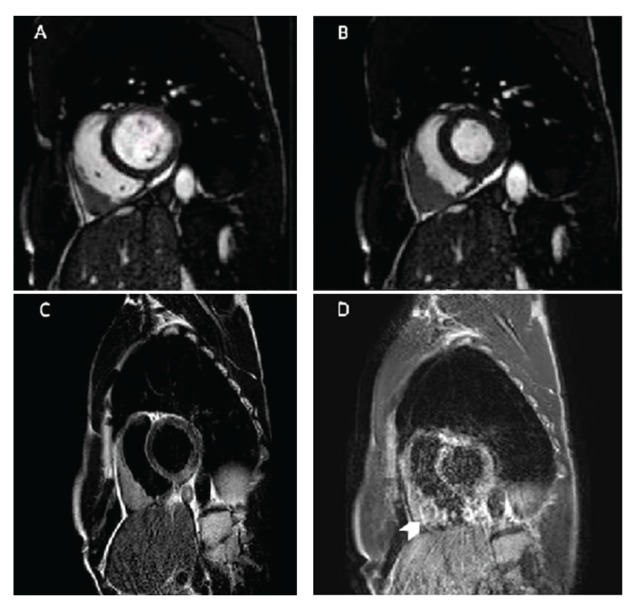
Short-axis diastolic (a) and systolic (b) phase clearly showing the large solid mass anteriorly to the right ventricle. (c) Short-axis T2w black-blood image easily depicting the hyperintense mass that largely infiltrates the myocardium of right ventricular wall which is thinned or completely undistinguishable from the pathological tissue. (d) Short-axis late gadolinium image showing the progressive ring enhancement of the lesion (arrow head), which was interpreted as a probable secondary malignant lesion.

Positron emission tomography/computed tomography (PET/CT), performed for restaging purpose, revealed the presence of a right angle cardiophrenic marked uptake (Figure 1). A cardiac magnetic resonance (CMR) study was performed to characterize this finding. CMR confirmed the presence of a solid mass (50 × 64 × 26 mm) inside the visceral paper of the pericardium and infiltrating the basal wall of the right ventricle (Figures 2(a) and 2(b)); this mass was characterized by a moderate hyperintensity on T2w images (Figure 2(c)) and by a progressive ring enhancement (Figure 2(d)) and was interpreted as a probable secondary malignant lesion. So the patient underwent cardiac surgery in order to obtain a histologic diagnosis of the ventricular mass and for debulking purpose. Surgical biopsy confirmed the secondary nature of the cardiac lesion histologically corresponding to a moderately differentiated adenocarcinoma, reported to be colorectal secondary malignancy, invading the sierosa with lymphovascular and perineural invasion. Unfortunately, debulking surgery could not be performed without sacrificing the tricuspid valve, since the metastasis deeply infiltrated it. Atrioventricular sulcus was affected by disease, as well.

The patient was then treated with FOLFOX-6 (leucovorin, fluorouracil, and oxaliplatin) chemotherapy regimen with poor results and, few months later, developed pericardial effusion, congestive heart failure, and syncopal attacks. Eventually, he died due to the obstruction of right ventricular outflow tract.

## 3. Discussion

Cardiac tumors are a peculiar subdivision of oncology. Although they are classified as either primary (benign or malignant) or secondary, we still lack a definitive classification based on aetiology, histopathology, or a system of staging for the tumors of the heart. Primary tumors of the heart are mostly myxomas whereas cardiac metastases result from lung, lymphoreticular, breast, and colon malignancies [2].

The incidence of cardiac metastases is probably underestimated. This is partly justified by the very low impact expected or because they are clinically silent in most cases. Indeed, few similar case reports [1, 3, 4] have been already documented. However, according to the literature, cardiac metastases have been found in 1,5%–20% of autopsies of cancer patients and in 0,2%–6,5% of subjects in unselected autopsy series [5, 6]. So why is the problem so underestimated in clinical practice? Is it possible that the incidence of cardiac metastases is really so low? This underestimation may be due to the inadequacy of routinely performed diagnostic modalities. In our case, CT scan alone could not reveal the presence of the pathological cardiac tissue, and the choice to perform a PET/CT and CMR, which easily identified it, was driven by the increase of tumor marker GICA. Nevertheless our patient turned out to be already beyond operability. For this reason, a more effective instrumental follow-up might be necessary at least for selected categories, such as young cancer patients with tumors known to frequently involve the heart, in order to early detect cardiac metastases. In such a context, treatment, although not curative, may lead to prolonged life expectancy and improved quality of life, which are valuable goals in this kind of patients.

There is not a standardized approach or treatment for patients with cardiac metastases because of the lack of randomized clinical trials, as well. So patients should be treated according to the established rules of the involved tumor [7].

However, several reports underline the role of surgery as the therapy of choice, as compared to chemotherapy; Murphy et al. performed operations on 19 patients with cardiac metastases and reported an operative survival of 68,4%, with a significant improvement of quality of life and prolonged life expectancy (5 patients had an average survival of 3.2 years) [8], as confirmed by other reports [3, 4].

In our case, since the patient was inoperable, chemotherapy was the only option we considered, but, unfortunately, he did not respond and died few months later.

So, despite the poor prognosis of patients with cardiac metastases, surgery, although not curative, should be preferred as compared to chemotherapy, especially in patients with localized disease or in presence of symptoms of obstruction.
